# Phytochemical and toxicological evaluation of Zephyranthes citrina

**DOI:** 10.3389/fphar.2022.1007310

**Published:** 2022-09-23

**Authors:** Muhammad Haseeb Ur Rehman, Uzma Saleem, Bashir Ahmad, Memoona Rashid

**Affiliations:** ^1^ Department of Pharmacology, Faculty of Pharmaceutical Sciences, Government College University, Faisalabad, Pakistan; ^2^ Department of Pharmacology, Hamza College of Pharmaceutical and Allied Health Sciences, Lahore, Pakistan; ^3^ Akhtar Saeed College of Pharmacy, Canal Campus Lahore, Lahore, Pakistan

**Keywords:** *Zephyranthes citrina*, UHPLC/MS, phytochemical analysis, toxicological study, LD_50_

## Abstract

Drugs obtained from medicinal plants have always played a pivotal role in the field of medicine and to identify novel compounds. Safety profiling of plant extracts is of utmost importance during the discovery of new biologically active compounds and the determination of their efficacy. It is imperative to conduct toxicity studies before exploring the pharmacological properties and perspectives of any plant. The present work aims to provide a detailed insight into the phytochemical and toxicological profiling of methanolic extract of *Zephyranthes citrina* (MEZ). Guidelines to perform subacute toxicity study (407) and acute toxicity study (425) provided by the organization of economic cooperation and development (OECD) were followed. A single orally administered dose of 2000 mg/kg to albino mice was used for acute oral toxicity testing. In the subacute toxicity study, MEZ in doses of 100, 200, and 400 mg/kg was administered orally, consecutive for 28 days. Results of each parameter were compared to the control group. In both studies, the weight of animals and their selected organs showed consistency with that of the control group. No major toxicity or organ damage was recorded except for some minor alterations in a few parameters such as in the acute study, leukocyte count was increased and decreased platelet count, while in the subacute study platelet count increased in all doses. In the acute toxicity profile liver enzymes Alanine aminotransferase (ALT), as well as, aspartate aminotransferase (AST) were found to be slightly raised while alkaline phosphatase (ALP) was decreased. In subacute toxicity profiling, AST and ALT were not affected by any dose while ALP was decreased only at doses of 200 and 400 mg/kg. Uric acid was raised at a dose of 100 mg/kg. In acute toxicity, at 2000 mg/kg, creatinine and uric acid increased while urea levels decreased. Therefore, it is concluded that the LD_50_ of MEZ is more than 2000 mg/kg and the toxicity profile of MEZ was generally found to be safe.

## 1 Introduction

Plants have historically been utilized as a fundamental source of new drugs. Herbal drugs have vast acceptability in the general population because of social beliefs regarding their excellent healing capacity, and the ability to improvise emotional well-being, thereby, augmenting the quality of life since ancient times (Aslam and Ahmad, 2016; Jamshidi et al., 2018). According to a report by the World Health Organization (WHO), nearly 80% population of low to middle-income countries (LMICs) is dependent on plant-based drugs to alleviate their primary health-related issues (Al-Nahain et al., 2014; Hoyler et al., 2018). Due to their myriad benefits, there is even a greater turnaround towards herbal remedies. These benefits include comparatively fewer adverse drug reactions whereas delivering promising outcomes (Muhammad I. et al., 2021). Moreover, certain reports state that herbal treatments trim down the pill burden (Rahimi et al., 2009). Plant-based drugs are used to treat various acute and chronic medical conditions such as parasitic infestation (Li Z et al., 2022), malaria, cancer (Majid M. et al., 2022), neurological disorders, age related degenerative disorders (Jin K. et al., 2022) chronic inflammation (Muhammad I. et al., 2022), cardiovascular and liver diseases, fungal (Chen H. et al., 2021) and bacterial infections (Yang L. et al., 2022), sleep disturbances such as insomnia, diabetes mellitus, and many others (Chandrasekaran & Venkatesalu, 2004; AlMamun, 2020). Medicinal plants provide cost-effectiveness and ease of use. Owing to their easy accessibility, inexpensive thrifty nature, and safety profile, the acceptability of herbal medicines has risen considerably, in recent times (Sandhya et al., 2006). Phytochemicals isolated from plant extracts have remained the mainstay of drug discovery (Muhammad I., and ul Hassan S.S., et al., 2021). Even in the present era of modern medicine, chemical compounds isolated from the plants are of particular interest because these compounds serve as a potential lead for newer drugs. Crude extracts of plants, as well as isolated phytochemicals, are screened for various *in vivo, in vitro*, and *in silico* pharmacological activities (Kundu P., et al., 2002). Plant extracts may possess both pharmacological and toxicological properties due to the presence of bioactive molecules (Yang J. et al., 2020). Extracts of various plants in different formulations, as well as isolated constituents, have widely been used as household remedies during the modern medicine era for the treatment of many diseases (Farnsworth, N. R. 1966).

Medicinal plants may contain toxic and pharmacologically active constituents. Some medicinal plants may intrinsically be toxic in terms of their phytochemicals which may be associated with adverse effects if used inadequately and improperly. Therefore, in the discovery of biologically active compounds (Zhuo, Z. et al., 2020), toxicity evaluation is the primary and mandatory parameter that needs to be assessed prior to its pharmacological screening and clinical application (Pour et al., 2011). Toxicity studies not only provide a correlation between the animal and human response by depicting the efficacy and safety profile but also help in ascertaining the dose of extracts for further screening (Anwar et al., 2021a). Thereby, toxicity studies safeguard the exposed population from the possible harms of the test compounds. Furthermore, these also help in appropriate dose assessment to be employed in end users (Mensah et al., 2019). Benefits of toxicological evaluation of plant extracts in animal models also include a controlled exposure time, examination of different tissues for possible harms, and determining the effect on different biomarkers (Arome and Chinedu, 2013). Conclusively, the toxicity study beneficially demarcates between toxic dose and therapeutic dose (Anwar et al., 2021b). Animal models are recommended for executing toxicological evaluations which comply with the organization of economic cooperation and development (OECD) guidelines.

Amaryllidaceae is a large family of plants that consists of 75 genera and 1,600 species (Christenhusz M.J.M. et al., 2016). Amaryllidaceae is famous for its alkaloids that have diverse biological activities. Numerous plants of Amaryllidaceae have traditionally been used as folklore medicine for the treatment of several ailments throughout the world (Biswas & Paul, 2022). *Zephyranthes citrina* (*Z. citrina*) is a naturally occurring perennial bulbous plant that belongs to Amaryllidaceae. Z. citrina has bright yellow flowers, green leaves, and bulbous stems. It is commonly known as Rain Lily because the flowering tops bloom in the rainy season (Jin and Yao, 2019). *Z. citrina* has been a relatively less explored plant for its pharmacological activities. A few pharmacological activities such as antimicrobial (Singh et al., 2010), antiprotozoal (Kaya et al., 2011), antimalarial (Herrera et al., 2001), anti-inflammatory (Aslam et al., 2016), and *in vitro* anticholinesterase activity which indicates its potential use in Alzheimer’s disease (Kohelová et al., 2021) have been reported so far.

Therefore, the present work aims at investigating the phytochemical and toxicity profiles of different doses of methanolic extracts of *Zephyranthes citrina* (MEZ) in order to report and identify the expected hazards in different doses using different protocols. The study has evaluated acute oral and subacute toxicity in mice models to ensure the safety and suitability of MEZ for further for its applicability in pharmacological screening.

## 2 Materials and methods

### 2.1 Chemicals

Methanol (I0962907) was purchased from Merck KGaA Germany. Pyrogallol solution was purchased from Oxford Labs (India). Other chemicals such as Carboxymethyl cellulose, sulfanilamide, Ethylenediamine tetraacetic acid (EDTA), Griess reagent, N-1- naphthylethyleneamine dihydrochloride, thiobarbituric acid, anhydrous aluminium chloride, copper sulfate, sodium phosphate dibasic heptahydrate and sodium phosphate monobasic monohydrate, phosphoric acid, picric acid, sodium carbonate, sodium hydroxide, sodium-potassium tartrate, gallic acid, Folin-Ciocalteu reagent, and quercetin were purchased from Sigma Aldrich United States.

### 2.2 Experimental validation

#### 2.2.1 Collection and authentication of plant material

The whole plant of *Z. citrina* was collected from the botanical garden of Government College University, Lahore (GCUL) Lahore during the flowering season from July to September. Plants have rush-like leaves and bulbous roots. Leaves bear yellow flowers that are Specific to *Z. citrina* within the entire family Amaryllidaceae. Authentication and validation of the plant were done by Prof. Dr. Zaheer Ud Din, botanist and taxonomist, at GCUL. The specimen of the plant was deposited in the Herbarium of the department of Botany GCUL vide voucher number (GC. Herb. Bot. 3553).

#### 2.2.2 Plant material preparation

Whole plants of *Z. citrina* were washed with tap water to remove debris (Saleem u et al., 2019). Leaves and flowers were separated from the bulbous part and air dried. Each bulb was cut into three slices and then dried in a hot air oven at 40°C until constant weight (López, S. et al., 2002). Once fully dried all parts of the plants were mixed together and ground by mechanical milling until a fine powder was obtained.

#### 2.2.3 Preparation of methanolic extract of *Z. citrina*

Powdered tissue of plant material (2 kg) was subjected to cold maceration, in the ratio of 1:2 with methanol (4 L), and was stirred every 8 h periodically, for 14 days. After the completion of the extraction period, initial filtration of the macerate was done through a filtration cloth to obtain the supernatant, separated from the macerated powder. This supernatant was subjected to subsequent filtration by passing it through a Buchner funnel assembly and Whatman filter paper number 1 under reduced pressure. This secondary filtration removed solid particulate matter suspended in the filtrate. Finally, a rotary evaporator was used for the evaporation of methanol from pure filtrate at 40°C under reduced pressure which yielded a dark brown gummy mass. The MEZ was kept in an airtight container between 2–8^°^C.

### 2.3 Estimation of total phenolic and flavonoid content

The Standard Folin-Ciocalteu reagent method was used for the determination of the total phenolic content (TPC) of MEZ (Terfassi, S, et al., 2021). Gallic acid was used as a standard for the determination of TPC. Briefly described, 1 ml of MEZ (final concentration 1 mg/ml) was mixed in 1 ml of Folin-Ciocalteu’s phenolic reagent. The solution was kept for 5 min and then 7% of Sodium carbonate (10 ml) was added and mixed. Then 13 ml of deionized distilled water was added to the previously made solution and thoroughly mixed for uniformity. This solution was incubated for one and a half hours in the dark at room temperature. After the incubation period, the absorbance was taken at 750 nm. Gallic acid solution was prepared and a calibration curve was constructed and extrapolated for the determination of the TPC of MEZ. The procedure was carried out in triplicate. Results were expressed as Gallic acid equivalents in milligram per Gram (GAE/g) of the dried sample (Saeed, N. et al., 2012).

For the determination of total flavonoid content (TFC) spectrophotometric method using anhydrous aluminium chloride (AlCl_3_ 6H_2_O) was used. Quercetin was employed as a standard.

Method followed is, 0.3 ml MEZ extract was mixed with 3.4 ml of 30% methanol, 0.15 ml of NaNO_2_ (0.5 M) and 0.15 ml of AlCl_3._6H_2_O (0.3 M). The solution was kept for 5 min and then 1 ml of sodium hydroxide was added to this solution. The whole solution was gently but thoroughly mixed and absorbance was measured at 506 nm. A standard curve was constructed using quercetin standard solution prepared by the above-mentioned procedure. The result for TFC was calculated and shown as milligram of quercetin equivalent per Gram of dried extract (mg QE/g) (Saeed, N. et al., 2012).

### 2.4 Antioxidant assay

*In vitro,* free radical scavenging activity was measured by a 2, 20 - diphenyl-1-picrylhydrazyl (DPPH) assay. The reaction of the DPPH assay depends on the capability of the plant extract samples to scavenge free radicals. The reaction is visually noticeable because the color changes from purple to yellow because of its ability to donate hydrogen. 24 mg DPPH was dissolved in 100 ml methanol to prepare the stock solution. This solution was stored at 20°C. The working solution was made by diluting DPPH with methanol until the final concert. Of DPPH becomes 0.267 mM in 0.004% methanol having an absorbance of almost 0.98 ± 0.02 at 517 nm by using the spectrophotometer. Then an aliquot of 1.5 ml was mixed in 50 μl of plant extract sample at varying concentrations between 10–500 μg/ml. This mixture was mixed thoroughly and incubated for 30 min in the dark at room temperature (Mishra, K. et al., 2012). The absorbance was recorded at 517 nm. The control solution was prepared by the same method mentioned above without any plant extract sample. Percentage DPPH scavenging activity was calculated by using Eq. 1 (Saeed, N. et al., 2012).

$$\text{Free}\;\text{Radical}\;\text{Scavenging}\;\text{Activity}\left(\%\right)=\frac{\text{Absorbance}\;\text{of}\;\text{control}-\text{Absorbance}\;\text{of}\;\text{sample}}{\text{Absorbance}\;\text{of}\;\text{control}}\times 100.$$ (1)

### 2.5 UHPLC–MS analysis for secondary metabolite profiling

Profiling of Secondary metabolites was carried out by reversed-phase ultra-high performance liquid chromatography-mass spectrometry (RP-UHPLC-MS) analytical technique. Agilent 1,290 Infinity ultra-high performance liquid chromatography system that has been attached to Agilent 6,520 Accurate–Mass Q-TOF mass spectrometer having dual electrospray ionization (ESI) source was used. Details and specifications of the column that was used are; Agilent Zorbax Eclipse XDB-C18 has a narrow bore size of 2.1 × 150 mm, 3.5 μm (P/N 930990-902). The temperature of 4°C for the auto-sampler and 25°C for the column was maintained. Two different mobile phase solutions were used A) 0.1% formic acid in water and B) 0.1% formic acid in acetonitrile. The mobile phase flow rate was kept at 0.5 ml/min 1.0 μl methanolic extract solution of the plant prepared in HPLC grade methanol was injected for 25 min And post-run time was 5 min. A complete scan of MS analysis using ESI negative ionization mode spanned over a complete range of *m/z* 100–1,000. Nitrogen was supplied both as nebulizing (flowrate 25 L/h) and drying gas (flowrate 600 L/h). The drying gas temperature was kept constant at 350°C. The capillary voltage was 3,500 V meanwhile fragmentation voltage had been optimized at 125 V. Data processing was done with Agilent mass hunter Qualitative Analysis B. 05.00, the Method used was Metabolomics −2017–0000.4 m (H. Saleem et al., 2019). Compound identification was done from the database: METLIN _AM_PCDL-N-170502. cdb with parameters as Match tolerance: 5 ppm. Positive Ions: +H, +Na, + NH_4_, negative Ions: H.

### 2.6 Experimental animals

Healthy adult Swiss albino mice were used as the experimental animals which were kept in the animal house of Government College University, Faisalabad, Pakistan. Standard controlled conditions were provided to the animals regarding temperature (22 ± 2°C) and humidity (45%–50%), 12 h light, and 12 h dark cycles having access to water and food, freely. Prior approval to conduct all the animal studies, including acute oral toxicity and subacute toxicity, was taken from Institutional Review Board, GC University Faisalabad vide letter number GCU/ERC/2141. Animals were treated ethically according to guidelines, rules, and regulations provided by the National Institute of Health (NIH, United States).

### 2.7 Toxicity studies protocols

#### 2.7.1 Acute oral toxicity

The OECD (Organization for Economic Corporation and Development) 425 guidelines (2001), were followed to conduct an acute oral toxicity study. Five healthy adult albino mice were used for acute oral toxicity tests. Animals were kept on fasting overnight with access to water *ad libitum*. Initially, only one animal, out of five, was administered 2,000 mg/kg body weight, as a single dose, of MEZ via an oral route through gastric lavage, and was subjected to observation for 24 h. If no mortality occurred to that animal, a single dose of 2,000 mg/kg of MEZ was administered, via the same route, to the remaining four animals. All the animals were observed for any signs of physical and behavioral alterations for 14 days

#### 2.7.2 Subacute toxicity

The OECD 407 guidelines (2008), with slight modification, were followed to conduct the subacute toxicity study. 40 healthy albino mice were used in the study. Animals were divided into four groups. Each group consisted of five females and five male animals. All animals, except the control, were administered different doses of extract (Table 1) for 28 days via an oral route through gastric lavage once daily. Physical conditions and behavioral aspects of animals were noted at the beginning of the experiment. Animals were observed daily for any change in weight and any sign of physical anomalies.

**TABLE 1:** Groups and doses of subacute toxicity study.

Group	Dose
1	Control (5–10 ml/kg Normal Saline)
2	MEZ^a^ 100 mg/kg of body weight
3	MEZ 200 mg/kg of body weight
4	MEZ 400 mg/kg of body weight

a MEZ: Methanolic Extract of *Z. citrina*.

#### 2.7.3 Weights of animals and their selected organs

During acute oral toxicity body weights of all the animals in all the groups were recorded at day zero, which was marked just before the start of the study, and then subsequently at days 1, 2, and 14. During the subacute study, body weights were measured on days 1, 7, 21, and 28. At the conclusion of the study, excision of the animals was carried out selected organs were separated and weighed.

### 2.8 Hematological parameter and biochemical markers measured in acute and subacute toxicity studies

After the completion of each study, animals were anesthetized by administering 5% isoflurane mixed with oxygen. A cardiac puncture was done for the collection of blood samples. Then these blood samples were subjected to hematological and biochemical analysis. Mindray BC3000 Plus hematology analyzer was used for hematologic analysis while for biochemical analysis Mindray BA88A was used. In regards to hematology following parameters were analyzed: Various aspects of platelet count, red blood cell (RBC) count, hematocrit, hemoglobin, mean corpuscular hemoglobin (MCH), mean corpuscular hemoglobin concentration (MCHC), total leukocyte count (TLC), and differential leukocyte count including both granulocytes and agranulocytes.

For the analysis of various biochemical markers, plasma and serum were prepared, separately. For the preparation of serum, a whole blood sample was coagulated at room temperature in a vacutainer and then it was centrifuged at a speed of 2,000 × g for 10 min. While plasma was prepared by collecting whole blood in an anticoagulant-containing vacutainer and then centrifuged at 2,000 × g for 10 min. Biochemical markers evaluated include liver function tests (LFTs), renal function tests (RFTs), and lipid profile (Liu C.et al., 2022). LFTs included AST, ALT, ALP, and protein. RFTs included urea, uric acid, creatinine, and bilirubin. The lipid profile included triglycerides (TGs), cholesterol, high-density cholesterol (HDL), and low-density cholesterol (LDL).

### 2.9 Histopathological studies

Selected organs such as the brain, heart, kidney, and liver were processed and preserved in a 4% formaldehyde solution. The organ specimens were embedded and fixed in paraffin wax and sections were made. Sliced sections were subsequently subjected to a fixation on slides and stained with hematoxylin and eosin (H & E) stain and histologically examined (Rajeh, M.A.B., 2012).

### 2.10 Observation of animal behavior and physical changes

All the animals were observed during acute for clinical signs of behavioral and physical alteration such as itching, eye, and nasal discharge, skin lesion, respiratory distress, abnormal movements and urination, and food and water intake. Any change in these parameters was recorded (Saleem H. et al., 2019).

### 2.11 Statistical analysis

GraphPad Prism 9.0 was used to interpret the data and expressed as standard deviation (±SD).

## 3 Results

### 3.1 Phytochemical composition and antioxidant activity

The methanolic extract of *Z. citrina* contains an abundant amount of phenolic and flavonoid compounds and was measured with reference to their standards and Gallic acid and Quercetin, respectively. Free radical scavenging activity by the DPPH method showed excellent antioxidant activity. Results for TPC, TFC, and antioxidant activity are mentioned in Table 2.

**TABLE 2:** Total content of bioactive compounds and antioxidant activity of MEZ.

Assay	Parameter	Results
Percent extract yield		19.7%
Content of bioactive compounds	Total flavonoid content	37.92 ± 0.26
	Total phenolic content	25.93 ± 0.19
Free radical scavenging activity	DPPH (%)	88.23 ± 2.95

#### 3.1.1 Secondary metabolite profiling (UHPLC–MS analysis)

**FIGURE 1:**
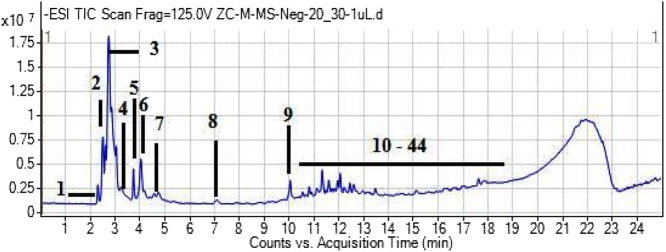
UHPLC - MS chromatogram of MEZ showing phytochemical profiling of extract.

UHPLC - MS Analysis of methanolic extract of *Z. citrina* was performed to determine the possible secondary metabolites and phytochemical components. The analysis showed that it contains 44 phytochemical compounds that belong to alkaloids, amino acids, carboxylic acids, flavonoids, phenolics, and a few other chemical classes. The phytochemical composition, retention time, base peak (*m/z*), chemical class, molecular mass, and formula are described in Table 3. The total ion chromatogram is shown in Figure 1.

**TABLE 3:** UHPLC-MS analysis of methanolic extract of Z. citrina.

S. No.	RT^a^ (Min.)	Base peak (m/z)	Molecular Mass	Proposed compound	Compound class	Molecular formula
1	2.294	272.9595	273.9647	Ribose-1-arsenate	Carbohydrate	C5 H11 As O8
2	2.474	241.0926	242.0998	D-erythro-D-galacto-octitol	Alditol	C8 H18 O S
3	2.485	173.1052	174.1125	L-Arginine	Amino Acid	C6 H14 N4 O S
4	2.501	224.0787	225.086	Acyclovir	Purine Analog	C8 H11 N5 O3
5	2.508	340.1261	341.1332	His Ala Asp	Amino Acid	C13 H19
6	2.522	335.1586	336.1648	N2-Fructopyra-nosylarginine	Phenolic	C12 H24 N4 O7
7	2.601	266.083	267.0902	PD 98059	Flavonoid	C16 H13 N O3
8	2.623	264.0987	265.1059	Agaritinal	Amino Acid derivative	C12 H15 N3 O4
9	2.649	219.0403	220.0479	Quinazoline acetic acid (3(2H)-Quinazolineacetic acid, 1,4-dihydro-2,4-dioxo-	Alkaloid	C10 H8 N2 O4
10	2.683	195.0519	196.0592	L-Gulonate	Carbohydrate	C6 H12 O7
11	2.686	165.0415	166.0488	1-Methylxanthine	Alkaloid	C6 H6 N4 O2
12	2.706	179.0573	180.0647	Theobromine	Alkaloid	C7 H8 N4 O2
13	2.747	683.2275	342.1186	Nigerose (Sakebiose)	Carbohydrate	C12 H22 O11
14	2.752	387.1165	388.1237	Fructoselysine 6-phosphate	Glycated protein	C12 H25 N2 O10 P
15	2.836	701.1932	666.2244	Maltotetraose	Carbohydrate	C24 H42 O21
16	2.843	539.1408	504.1714	Panose	Carbohydrate	C18 H32 O16
17	2.891	827.2702	828.2771	Maltopentaose	Carbohydrate	C30 H52 O26
18	2.921	989.3229	990.3299	Maltohexaose	Carbohydrate	C36 H62 O31
19	2.926	369.1042	370.1118	2′,3′,5′-triacetyl-5-Azacytidine	Pyrimidine nucleoside analogue	C14 H18 N4 O8
20	2.95	1,151.374	1,152.381s	Celloheptaose	Sugar	C42 H72 O36
21	2.966	149.0459	150.0532	L-Lyxose	Aldehyde	C5 H10 O5
22	2.973	339.0972	304.1293	2′-Deoxymugineic acid	Carboxylic acid	C12 H20 N2 O7
23	2.978	366.1165	367.124	Met Ser Met	Protein	C13 H25 N3 O5 S2
24	3.093	133.015	134.0225	3,3-Dimethyl-1,2-dithiolane	Dithiolanes	C5 H10 S2
25	3.259	290.0886	291.0959	Sarmentosin epoxide	Glycoside	C11 H17 N O8
26	3.939	191.0202	192.0275	Citric acid	Carboxylic acid	C6 H8 O7
27	4.026	128.0359	129.0431	N-Acryloylglycine	Amino acid	C5 H7 N O3
28	4.164	243.0624	244.0698	Uridine	Pyrimidine Nucleoside	C9 H12 N2 O6
29	4.403	130.0873	131.0945	L-Leucine	Amino acid	C6 H13 N O2
30	4.534	180.0666	181.0738	3-Amino-3-(4-hydroxyphenyl) propanoate	Amino acid	C9 H11 N O3
31	4.58	292.1404	293.1476	N-(1-Deoxy-1-fructosyl) leucine	Leucine & derivative	C12 H23 N O7
32	4.729	288.1242	289.1314	Norcocaine	Alkaloid	C16 H19 N O4
33	4.828	103.0403	104.0475	D (-)-β-hydroxy butyric acid	Carboxylic acid	C4 H8 O3
34	10.042	255.0519	256.0591	Piscidic Acid	Phenolic	C11 H12 O7
35	10.536	153.0197	154.0269	3,4-Dihydroxybenzoic acid	Phenolic	C7 H6 O4
36	10.887	385.0568	386.0645	Shoyuflavone A	Flavanoids	C19 H14 O9
37	11.228	175.0613	176.0685	3-propylmalic acid	Carboxylic acid	C7 H12 O5
38	11.325	239.0563	240.0636	(1R,6R)-6-Hydroxy-2-succinylcyclohexa-2,4-diene-1-carboxylate	Gamma keto acid	C11 H12 O6
39	11.382	215.0827	216.09	Desethyletomidate	Ethylester	C12 H12 N2 O2
40	11.573	183.03	184.0373	4-O-Methyl-gallate	Phenolic	C8 H8 O5
41	11.948	306.0619	307.0692	Narciclasine	Alkaloid	C14 H13 N O7
42	11.949	352.067	353.0745	2,5-Diamino-6-hydroxy-4-(5′-phosphoribosylamino)-pyrimidine	N-glycosyl	C9 H16 N5 O8 P
43	13.461	187.0977	188.105	Nonic Acid	Pyruvic Acid	C9 H16 O4
44	17.614	293.1765	294.1838	Gingerol	Phenolic	C17 H26 O4

a RT: retention time.

### 3.2 Weights of animals and their selected organs in acute and subacute toxicity

**FIGURE 2:**
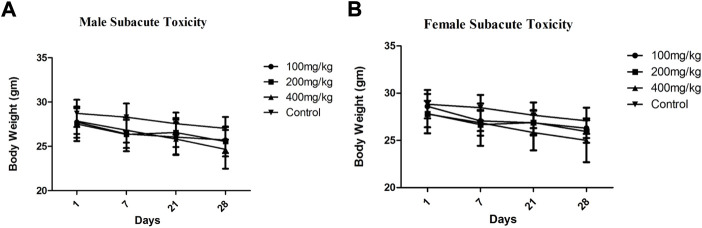
Average body weights ±SD of Male **(A)** and female **(B)** animals treated at different doses along with control during subacute toxicity study.

**FIGURE 3:**
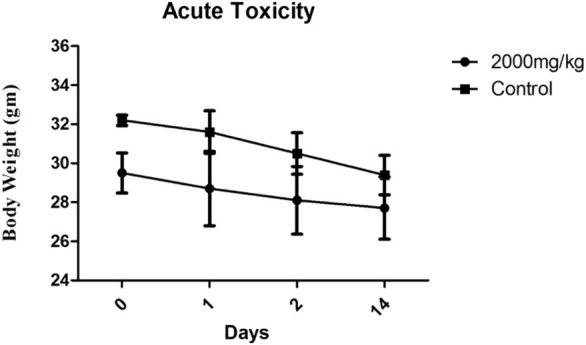
Average body weights ±SD of treated animals at 2,000 mg/kg dose along with control group during acute toxicity study.

During both the studies body weights were monitored and recorded are described in Table 4 and Figure 2. The average body weights with a standard deviation of all treated and control animals during the subacute toxicity study of both male and female groups were observed from the day 1 to day 28th (Figures 2A,B respectively). MEZ did not show any considerable difference in body weights of animals from day 1–28 in comparison to control. The average body weights with SD of animals treated at 2,000 mg/kg and control group animals during the acute toxicity study were observed and mentioned in Figure 3. The acute toxicity study which was carried out on days 0, 1, 2, and 14 showed no major change in body weights of the treated group in comparison to control group (Figure 3).

**TABLE 4:** Selected organ weights with standard deviation of treated and control group animals during acute and subacute toxicity Study.

	Dose (mg/kg)	Liver	Kidney	Pancreases	Lungs	Heart	Stomach	Brain
Acute Toxicity	**2000**	1.56 ± 0.09	0.43 ± 0.06	0.12 ± 0.018	0.26 ± 0.0 4	0.14 ± 0.02	0.25 ± 0.02	0.39 ± 0.017
	**Control**	1.53 ± 0.05	0.41 ± 0.03	0.14 ± 0.03	0.24 ± 0.04	0.12 ± 0.01	0.24 ± 0.014	0.40 ± 0.02
Subacute Toxicity (Male)	**100**	1.30 ± 0.03	0.44 ± 0.03	0.12 ± 0.01	0.27 ± 0.01	0.13 ± 0.003	0.23 ± 0.01	0.42 ± 0.02
	**200**	1.15 ± 0.08	0.35 ± 0.03	0.11 ± 0.18	0.21 ± 0.01	0.13 ± 0.003	0.22 ± 0.02	0.40 ± 0.01
	**400**	1.58 ± 0.06	0.42 ± 0.04	0.15 ± 0.02	0.29 ± 0.02	0.14 ± 0.01	0.24 ± 0.01	0.37 ± 0.03
	**Control**	1.57 ± 0.03	0.42 ± 0.03	0.14 ± 0.01	0.22 ± 0.01	0.12 ± 0.01	0.23 ± 0.012	0.39 ± 0.015
Subacute Toxicity (Female)	**100**	1.46 ± 0.04	0.42 ± 0.03	0.13 ± 0.01	0.26 ± 0.01	0.13 ± 0.04	0.22 ± 0.01	0.4 ± 0.02
	**200**	1.47 ± 0.08	0.37 ± 0.03	0.10 ± 0.018	0.22 ± 0.01	0.137 ± 0.008	0.23 ± 0.02	0.4 ± 0.017
	**400**	1.68 ± 0.09	0.42 ± 0.02	0.15 ± 0.01	0.28 ± 0.02	0.13 ± 0.01	0.24 ± 0.02	0.39 ± 0.03
	**Control**	1.46 ± 0.08	0.34 ± 0.04	0.13 ± 0.02	0.25 ± 0.03	0.12 ± 0.009	0.24 ± 0.03	0.38 ± 0.03

Organ weights, measured at the completion of subacute and acute oral toxicity studies, are described in Table 4. Organ weights of MEZ-treated animals in both studies were comparable to the control groups which depicts that MEZ is not involved in organ damage.

### 3.3 Hematological analysis in subacute and acute oral toxicity studies

Blood samples were collected at the end of both studies through cardiac puncture and were subjected to CBC analysis. During the acute oral toxicity study, WBCs were raised significantly at the dose of 2,000 mg/kg in comparison to control group animals. Upon detailed analysis, granulocyte count was also found to be raised. Erythrocyte count was normal in the treatment group while the platelet count was decreased in comparison to the control group. Results of hematological parameters of acute oral toxicity study are shown in Table 5.

**TABLE 5:** Complete blood count of treated and control group animals during acute oral toxicity study.

Dose	Complete blood count (CBC)														
	WBC	LYM	MID	GRA	LYM	MID	GRA	RBC	HGB	MCV	HCT	MCH	MCHC	RDWsd	RDWcv
	103/µL	103/µL	103/µL	103/µL	%	%	%	106/µL	g/dL	fL	%	pg	g/dL	fL	%
2000 mg/kg	26.50 ± 2.27	4.58 ± 0.07	1.53 ± 0.26	0.74 ± 0.1	80.6 ± 0.20	18.9 ± 0.20	12.61 ± 0.24	8.59 ± 0.25	14.5 ± 0.5	52 ± 0.35	45.76 ± 0.29	17.83 ± 0.29	31.93 ± 0.51	27.20 ± 0.36	17.16 ± 0.31
CONTROL	7.19 ± 0.20	6.92 ± 0.09	0.66 ± 0.04	0.23 ± 0.07	90.21 ± 0.16	52.3 ± 0.18	2.32 ± 0.1	8.28 ± 0.49	13.37 ± 0.34	48.9 ± 0.75	41.08 ± 0.25	16.08 ± 0.66	32.80 ± 0.61	28.07 ± 0.21	18.97 ± 0.42
	PLT			MPV		PCT		PDWsd		PDWcv		PLC-R		PLC-C	
	103/µL		fL		%		fL		%		%		103/µL	
2000 mg/kg	375.67 ± 6.51		7.13 ± 0.15		0.68 ± 0.03		31.31 ± 0.14		44.0 ± 0.75		22.3 3 ± 0.58		215 ± 4.58	
CONTROL	594.00 ± 3.61		7.26 ± 0.10		0.41 ± 0.02		8.41 ± 0.31		47.93 ± 0.31		23.90 ± 0.38		140.23 ± 0.21	

During the subacute study of both male and female animals, CBC analysis showed no major change in any WBC and RBC count in all treatment groups of both genders. Platelet count was relatively higher in both male and female mice of the treatment groups than in the control group. Results of both male and female animals are shown in Table 6.

**TABLE 6:** Complete blood count of treated and control group animals during subacute toxicity study.

Dose (mg/kg)	Male							Female						
WBC’s	WBC	LYM	MID	GRA	LYM	MID	GRA	WBC	LYM	MID	GRA	LYM	MID	GRA
	103/µL	103/µL	103/µL	103/µL	%	%	%	103/µL	103/µL	103/µL	103/µL	%	%	%
100	5.43 ± 0.15	3.9 ± 0.01	1.07 ± 0.12	0.623 ± 0.08	72.3 ± 0.20	17.3 ± 0.40	10.33 ± 0.15	5.40 ±	3.90 ±	1.00 ±	0.60 ±	72.10 ±	17.70 ±	10.20 ±
200	5.503 ± 0.06	4.78 ± 0.02	0.58 ± 0.04	0.21 ± 0.1	85.46 ± 0.4	10.8 ± 0.46	3.343 ± 0.06	5.50 ±	4.80 ±	0.60 ±	0.20 ±	85.90 ±	10.70 ±	3.40 ±
400	5.63 ± 0.32	2.55 ± 0.06	2.77 ± 0.08	0.31 ± 0.04	43.36 ± 1.30	50.5 ± 0.50	5.58 ± 0.080	5.90 ±	2.61 ±	2.70 ±	0.31 ±	43.80 ±	50.50 ±	5.65 ±
CONTROL	6.18 ± 0.13	5.34 ± 0.06	0.6 ± 0.02	0.11 ± 0.02	88.16 ± 0.25	48.3 ± 0.1	2.09 ± 0.04	6.33 ±	5.35 ±	0.58 ±	0.11 ±	87.90 ±	9.46 ±	2.13 ±

RBC	RBC	HGB	MCV	HCT	MCH	MCHC	RDWsd	RDWcv	RBC	HGB	MCV	HCT	MCH	MCHC	RDWsd	RDWcv
	106/µL	g/dL	fL	%	pg	g/dL	fL	%	106/µL	g/dL	fL	%	pg	g/dL	fL	%
100	8.74 ± 0.25	14.80 ± 0.52	52.20 ± 0.34	45.60 ± 0.29	17.00 ± 0.28	32.50 ± 0.51	27.50 ± 0.36	17.50 ± 0.31	8.31 ± 0.27	13.90 ± 0.48	51.60 ± 0.30	46.10 ± 0.29	16.50 ± 0.26	31.50 ± 0.48	26.80 ± 0.33	16.90 ± 0.29
200	9.31 ± 0.33	14.10 ± 0.31	47.53 ± 0.27	44.37 ± 0.31	16.07 ± 0.29	32.23 ± 0.47	31.60 ± 0.35	22.23 ± 0.26	9.32 ± 0.23	13.90 ± 0.51	46.90 ± 0.28	44.30 ± 0.27	15.90 ± 0.21	32.90 ± 0.43	31.90 ± 0.29	21.90 ± 0.28
400	6.12 ± 0.29	10.70 ± 0.26	46.40 ± 0.30	33.10 ± 0.28	15.90 ± 0.27	33.90 ± 0.36	31.10 ± 0.33	22.10 ± 0.27	6.86 ± 0.26	11.20 ± 0.47	47.20 ± 0.29	32.40 ± 0.31	16.40 ± 0.29	34.70 ± 0.41	30.40 ± 0.31	21.40 ± 0.26
CONTROL	8.28 ± 0.26	13.37 ± 0.25	48.92 ± 0.21	41.08 ± 0.27	16.08 ± 0.30	32.80 ± 0.34	28.07 ± 0.35	18.97 ± 0.28	8.53 ± 0.26	13.70 ± 0.51	49.00 ± 0.31	41.80 ± 0.26	16.10 ± 0.15	32.90 ± 0.37	28.20 ± 0.28	19.20 ± 0.28

Platelets	PLT	MPV	PCT	PDWsd	PDWcv	PLC-R	PLC-C	PLT	MPV	PCT	PDWsd	PDWcv	PLC-R	PLC-C
	103/µL	fL	%	fL	%	%	103/µL	103/µL	fL	%	fL	%	%	103/µL
100	976.00 ± 165.50	7.00 ± 0.13	0.68 ± 0.31	7.90 ± 1.62	44.80 ± 7.82	22.00 ± 3.32	211.00 ± 20.34	982.00 ± 170.64	7.30 ± 0.42	0.72 ± 0.37	8.20 ± 1.20	43.30 ± 6.39	23.00 ± 2.29	214.00 ± 38.36
200	>1,000 ± 132.76	7.90 ± 0.34	0.80 ± 0.24	9.50 ± 0.96	48.20 ± 8.92	32.00 ± 4.51	324.00 ± 31.24	1,002.00 ± 210.21	7.40 ± 0.69	0.84 ± 0.64	9.32 ± 0.92	47.50 ± 7.23	31.00 ± 3.79	319.00 ± 41.36
400	815.00 ± 210.20	9.10 ± 0.45	0.83 ± 0.34	12.20 ± 0.94	51.20 ± 10.2	35.80 ± 3.98	376.00 ± 52.61	792.00 ± 118.85	9.90 ± 1.20	0.80 ± 0.72	12.50 ± 1.04	50.20 ± 8.02	46.00 ± 8.41	372.00 ± 36.41
CONTROL	597.00 ± 314.24	7.33 ± 0.52	0.43 ± 0.31	8.19 ± 1.26	48.00 ± 7.92	24.00 ± 3.26	340.00 ± 34.2	595.00 ± 206.39	7.15 ± 0.83	0.40 ± 0.36	8.25 ± 1.14	48.20 ± 7.05	23.48 ± 3.92	348.10 ± 45.92

### 3.4 Biochemical markers in acute and subacute studies

**FIGURE 4:**
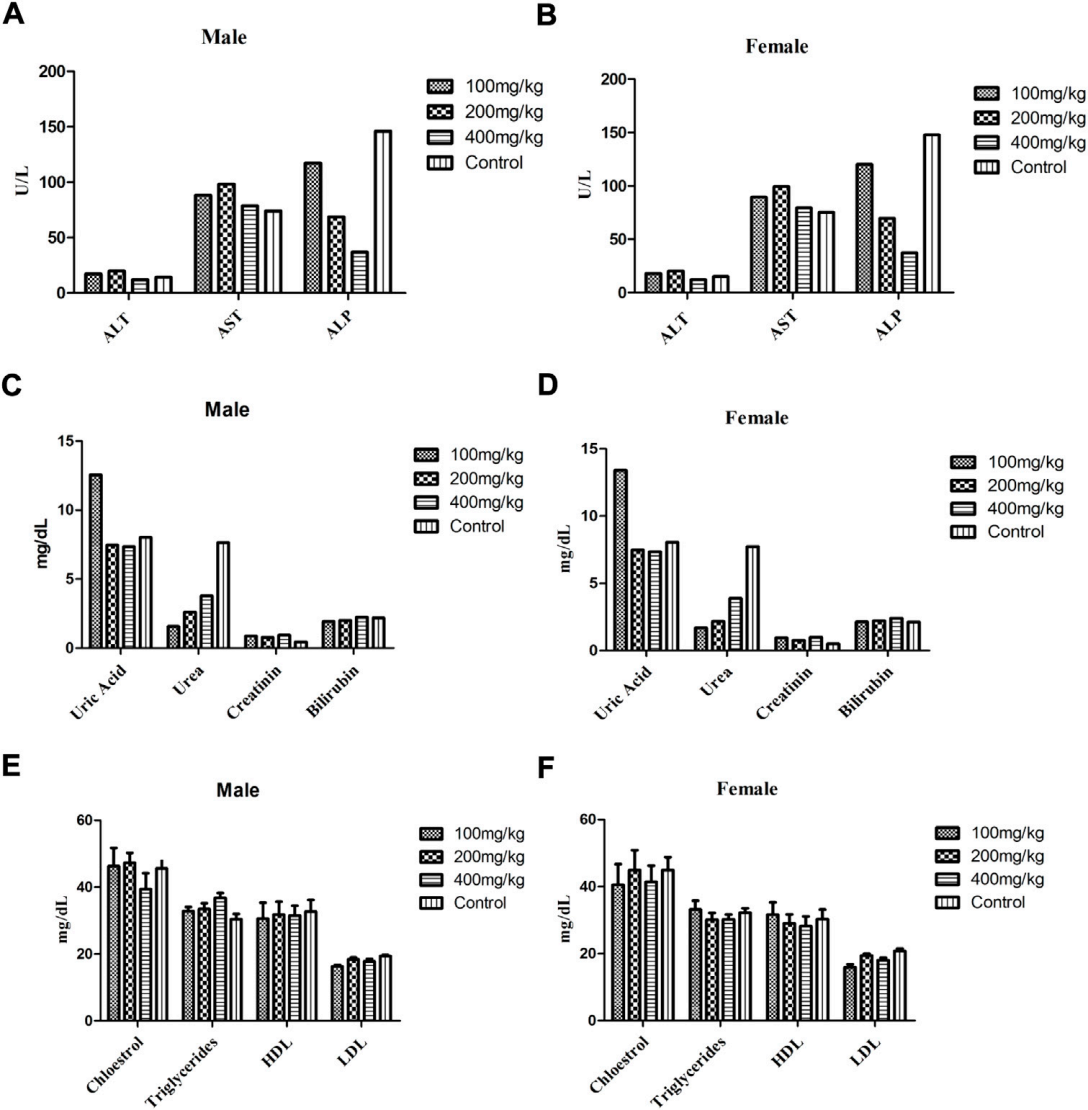
Biochemical markers with ±SD in both male and female animals of treatment and control group during subacute study, LFTs **(A,B)**, RFTs **(C,D)** and total lipid profile **(E,F)**, respectively.

Figure 4 and Table 7 describe the results of subacute and acute studies respectively of various biochemical markers such as LFTs (AST, ALT, ALP, and protein), RFTs (Uric acid, urea, creatinine, and bilirubin), and total lipid profile (cholesterol, triglycerides, HDL, and LDL). During the subacute study, there was no change in levels of AST, and ALT in all treated groups while the level of ALP was decreased at doses of 200 and 400 mg/kg in treated groups. The level of ALP in animals treated at a dose of 100 mg/kg remained comparable to the control group. During the acute toxicity study in the treatment group levels of AST and ALT were raised while that of ALP was decreased in comparison to control. Results of LFTs for both male and female animals’ subacute study are shown in Figures 4A,B, respectively.

**FIGURE 5:**
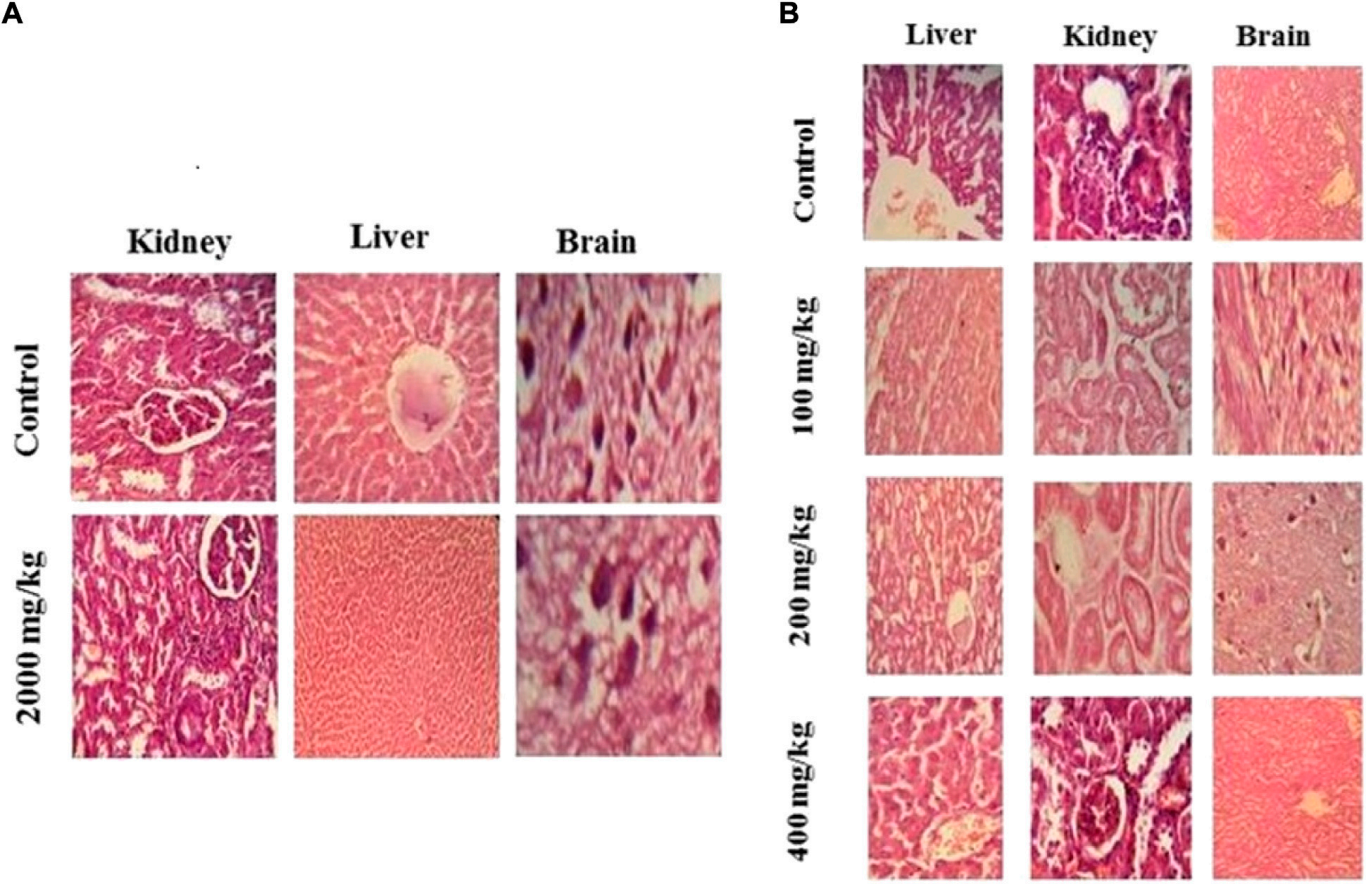
H & E stained histopathological analysis of selected organs. Acute oral toxicity study **(A)** and subacute toxicity study **(B)**.

**TABLE 7:** Biochemical marker analysis after treatment of 2000 mg/kg and control group during acute toxicity study.

Biochemical marker	Unit	2000 mg/kg	Control
ALT	U/L	28.9 ± 2.15	14 ± 1.43
AST	U/L	100.5 ± 3.62	74 ± 2.7
ALP	U/L	119.5 ± 10.57	145 ± 6.69
Uric Acid	mg/dL	12.43 ± 0.19	8.13 ± 0.08
Urea	mg/dL	1.6 ± 0.36	7.7 ± 0.22
Creatinine	mg/dL	0.82 ± 0.05	0.46 ± 0.1
Protein	g/dL	15.4 ± 0.38	13.13 ± 0.20
Bilirubin	mg/dL	1.91 ± 0.22	2.24 ± 0.15
Cholesterol	mg/dL	44.73 ± 4.68	48.23 ± 3.42
Triglycerides	mg/dL	37.92 ± 1.73	35.36 ± 1.56
HDL	mg/dL	27.32 ± 3.72	30.22 ± 2.58
LDL	mg/dL	12.34 ± 0.78	15.94 ± 0.64

RFT (Uric acid, urea, creatinine, and bilirubin) analysis at the end of the subacute study of treated animals at all doses showed comparable results for bilirubin and creatinine in both genders in comparison to the control group. The levels of uric acid were higher in the treatment groups at doses of 100 mg/kg in comparison to other treatment and control groups. The level of urea was less in all treatment groups other than the control group. The RFT profile showed similar results regardless of gender (Figures 4C,D). During the acute toxicity study levels of creatinine and uric acid were higher, while the levels of urea were less in the treatment group as compared to the control group. The level of bilirubin and total protein was comparable in the control and treatment groups (Table 7).

Total lipid profile analysis showed no significant difference in any treatment group at any dose during the subacute and acute toxicity study. Figures 4E,F describe the total lipid profile in male and female animals respectively of all treated and control groups. Results of the lipid profile of the acute toxicity study are shown in Table 7.

### 3.5 Histopathology analysis

Histopathologic examination of slides revealed no major toxicity concern in the morphology of selected organs. Brain, kidney, liver, and heart tissue showed normal morphological features in all treatment groups in acute and subacute toxicity studies. No signs of necrosis were observed in any tissue in any treatment group. However, the liver showed slight cytoplasmic ballooning and fatty tissue deposit at various doses in the subacute toxicity studies.

### 3.6 Animal behavior and physical changes

During the acute oral toxicity study, all animals that were treated with 2,000 mg/kg of MEZ remained healthy and showed no clinical signs of any abnormality in observed parameters. Results are shown in Table 8.

**TABLE 8:** Effect of test doses of MEZ on clinical signs of behavioral and physical parameters in the acute oral toxicity study.

Clinical parameter	Control	2,000 mg/kg
Itching	-	-
Eye discharge	-	-
Nasal discharge	-	+
Skin lesion	-	-
Respiratory distress	-	-
Abnormal movement	-	-
Urination	Normal	Normal
Food intake	Normal	Normal
Water Intake	Normal	Normal

No clinical sign (-), mild clinical sign present (+), Moderate to Severe sign present (++).

## 4 Discussion

Drugs obtained from natural sources play an imperative role in the field of medicine and in the development of novel agents, as well. The ethnobotanical knowledge could be helpful in serving mankind by conducting new research and exploring novel drug products. In parallel to the discovery of new biologically active compounds and determination of their efficacy, safety profiling of these compounds and plant extracts is of utmost importance. Many regulations are in place for prior pre-clinical studies regarding the safety of new compounds (Aslam and Ahmed, 2016). To ensure the safety of humans from the lethal effects of the test compounds, a toxicological evaluation of the test compound is carried out which follows standard protocols set forth by regulatory bodies (Anwar et al., 2021b). The protocols strictly emphasize the safety of the human population which regulates the laws regarding the toxicological evaluation of all test compounds prior to their approval. It further comprises the administration of single and repeated doses and requires different evaluations on both genders of animals. This serves as the basis of various guidelines for acute oral and subacute toxicological evaluation in animal models. Oral acute toxicity is conducted with the single maximum dose of 2000 mg/kg in order to explicit any deleterious effects from the test compound while subacute toxicity is carried out by the repeated doses of the test compound for 28 days to study their impact or effect on any abnormality in various predefined parameters (Klaassen and Amdur, 2013; Abubakar et al., 2019).

An acute oral toxicity study is also crucial in determining the LD_50_ of unknown extracts and phytochemicals. This dose determination at the preclinical stage is helpful in determining the safety margin of the test compound. It also provides information about at what doses further pharmacological screening could be carried out (Mohs and Greig, 2017). Therefore, to assess the phytochemical composition, MEZ was subjected to UHPLC–MS analysis. The LC/MS analysis determined the presence of a diverse array of chemicals in MEZ which include alkaloids, phenols and flavonoids, and carbohydrates. Although most of the constituents can be found in the scientific literature many of these have not been reported previously in Z. citrina described in Table 4 (Kohelová et al., 2021; Biswas and Paul, 2022). MEZ showed excellent free radical scavenging and antioxidant activity by the DPPH method (88%). The antioxidant activity of MEZ is possibly due to the presence of diverse antioxidant phytochemicals such as flavonoids, and phenolic compounds. The possible phytochemicals that may be responsible for antioxidant activity present in MEZ are methyl gallate and gingerol. Enzymes that are involved in antioxidant activities are upregulated by methyl gallate. Therefore, methyl gallate protects different tissues, for example, the heart, neurons, adipose tissue, hepatocytes, RBCs, and renal cells against the deleterious effects of toxic compounds. Methyl gallate also possesses anti-HIV properties as well (Ng, T. B. et al., 2018). Gingerol has free radical scavenging activity and therefore inhibits lipid peroxidation and acts as an antioxidant (Masuda, Y. et al., 2004).

Due to the presence of multiple arrays of phytochemicals in MEZ, its acute oral and subacute toxicity study was also conducted to assess implications on various hematological, biochemical and behavioral parameters because any variation in body and organ weights, hematological parameters, and biochemical markers can provide a piece of substantial evidence for the toxicological profiling of plant extracts (Variya et al., 2019). MEZ was subjected to toxicity testing through oral acute toxicity and subacute toxicity in Swiss albino mice. OECD guidelines for both studies were used. With the assumption of the test compound is nontoxic, the limit test was performed (Prabu et al., 2013). This means the test compound has been evaluated at the highest dose of 2000 mg/kg which also identifies a lethal dose (Bhattacharya et al., 2011). Outcomes revealed that no cases of mortality and morbidity were found upon MEZ administration at 2,000 mg/kg dose which declares it nontoxic at 2,000 mg/kg. The subacute toxicity of MEZ was assessed for 28 days at different doses of 100, 200, and 400 mg/kg of body weight. These variations may be induced by the test compound or its metabolites. As the results indicated (Table 4) no noticeable alterations in body weight or organ weights (liver, kidney, pancreas, lungs, heart, stomach, and brain) and no lethal effects were found throughout both the studies. No alterations in the behavioral parameters of animals were found (Table 8). Likewise, neither mortality nor morbidity was seen throughout the test period which was depicted by all animals in both studies did not show signs of weight loss. During toxicity studies, a considerable change in the total body weight of animal or organ weight is attributed to unfavorable physiological (food intake, stress, and diurnal changes) or pathological events (immunomodulation) (Dybing et al., 2002) which show that MEZ is non-toxic. Therefore, the MEZ is found to be safe at tested doses and the oral LD_50_ was considered to be greater than 2000 mg/kg in mice.

Blood dyscrasias or alterations of hematological parameters could also indicate toxicity of the plant extracts. All the blood corpuscles originate from uncommitted pluripotent hematopoietic stem cells within the bone marrow. These stem cells upon appropriate signals are converted into committed pluripotent hematopoietic stem cells. These committed cells then produce colony-forming units (CFUs) and individual blood cells are formed (Broxmeyer, H. E., 1988). Regarding hematological parameters, MEZ at 2,000 mg/kg dose elevated the level of granulocyte count while there was a slight decrease in platelet count. The slight increase in granular leukocytes may be attributed to immunomodulation (Anwar, F. et al., 2021). No considerable change was observed in the subacute study at different doses in WBCs and RBCs. Only the platelet count was found a bit higher at all doses than the control value in both male and female mice. This might be attributed to the immuno-stimulatory behavior, on bone marrow, expressed by MEZ and different phytochemicals such as phenols and flavonoids may be responsible for this (Onuh, S. N., 2012). All other parameters were either normal or with slight variations but well within the normal limits.

Assessment of liver and kidney functions is considered vital for the toxicity profiling of plant extracts. Therefore, liver function tests (LFTs) and renal function tests (RFTs) may greatly indicate the signs of toxicity induced by plant extracts. The liver is majorly connected with the metabolism while the kidneys are related to the excretion of elimination of drug substances. Findings of acute toxicity studies revealed the elevation in ALT and AST levels at the dose of 2,000 mg/kg with the decline in ALP level. An increase in ALT level indicates hypertrophy, as it is the sole sensitive biomarker for liver functioning while a high AST level is associated with hepatic damage at the cellular level and ALP, symbolizes biliary function (Ozer et al., 2008; Anwar et al., 2021a). Histologic evaluation of liver tissue showed mild ballooning of cytoplasm and mild fatty tissue deposition which correlates to an increase in AST level. The study did not report any unusual change in RFT except a slight increase in uric acid which may be due to interference with uric acid metabolism or decreased urate excretion (Asiwe, J. N., 2022). Histologic evaluation showed normal morphological features of the kidney which excludes any kidney damage. The lipid profile was measured by cholesterol, triglyceride, HDL, and LDL levels. No considerable variation was observed on the lipid profile of MEZ over treated mice with a meager elevation on triglycerides only which shows good tolerability of MEZ over a wide range of doses and depicts its safety. Safety profile of MEZ is well enough to create any harm at the cellular or molecular level. Thus the MEZ emerged safe to be used in the medicinal field for its pharmacological activities.

## 5 Conclusion

Conclusively the hematological, and biochemical markers, histological study, and other physical observations confirm the safety and tolerability of MEZ with minor and insignificant alterations in a few parameters. A mild increase in uric acid shows interference with uric acid metabolism or excretion. Histologic evaluation liver showed slight oxidative stress on liver tissue which was confirmed by an increased level of AST in mice treated at doses of 2,000 mg/kg in the acute toxicity study. The LD_50_ of MEZ is greater than 2,000 mg/kg. MEZ is safe at tested doses and can be explored for any future pharmacological evaluation in the mice model. Yan et al., 2020.

## Data Availability

The raw data supporting the conclusions of this article will be made available by the authors, without undue reservation.
