# Supplementary figures and images for: Phytochemical and toxicological evaluation of Zephyranthes citrina
Source: Front Pharmacol. 2022 Sep 23;13:1007310. doi: 10.3389/fphar.2022.1007310 (PMC9539839; doi:10.3389/fphar.2022.1007310)

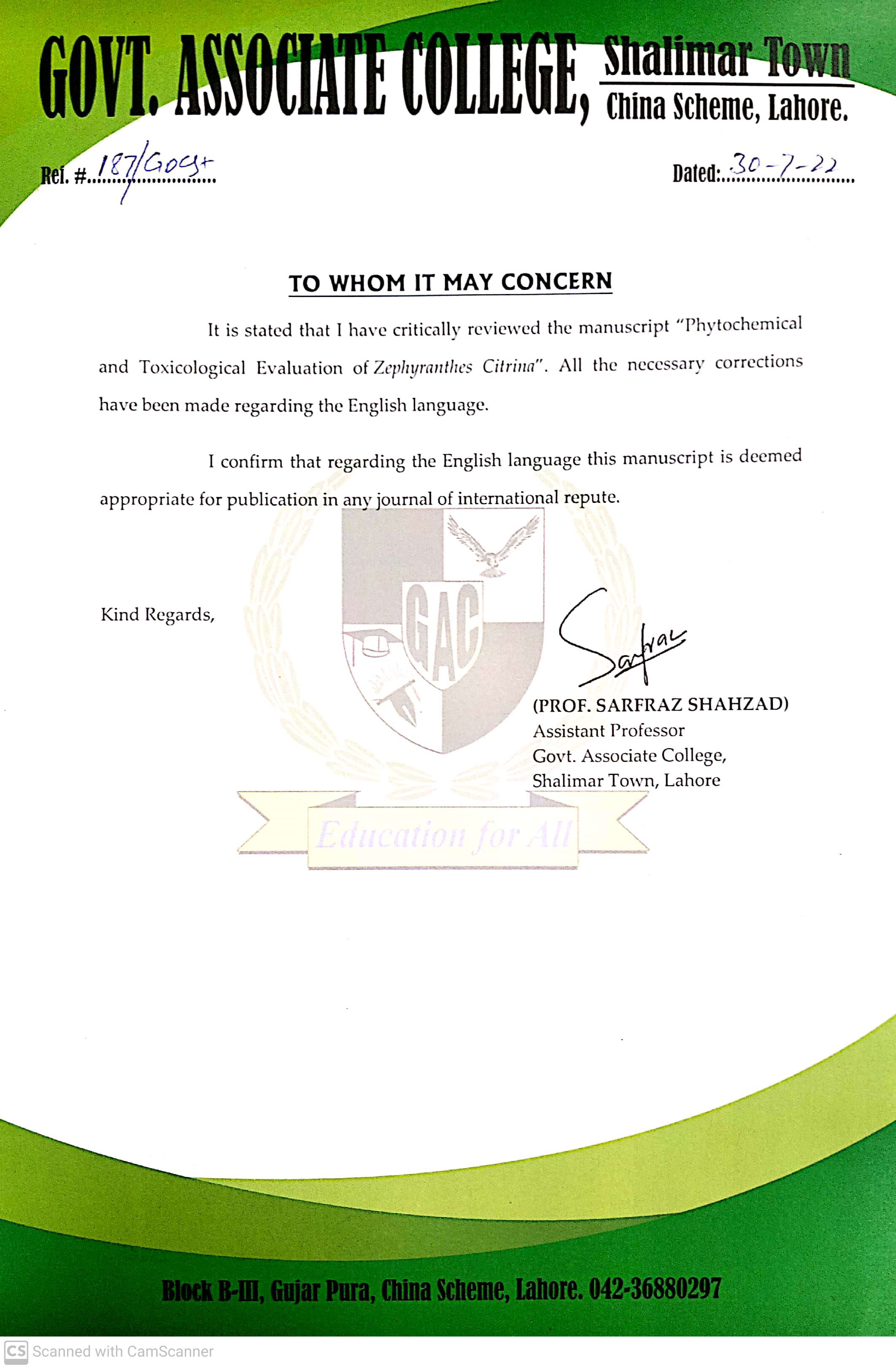

Supplement: Supplementary file 1 [file Image1.JPEG]
